# Aerotolerance and Multi-Locus Sequence Typing of *Campylobacter jejuni* Isolated from Commercial Broiler Processing Plants

**DOI:** 10.3390/foods12173305

**Published:** 2023-09-02

**Authors:** Diksha Pokhrel, Hudson T. Thames, Li Zhang, Thu Dinh, M. Wes Schilling, Shecoya White, Reshma Ramachandran, Anuraj T. Sukumaran

**Affiliations:** 1 Department of Poultry Science, Mississippi State University, Mississippi State, MS 39762, USA; dp1606@msstate.edu (D.P.); htt37@msstate.edu (H.T.T.); lz245@msstate.edu (L.Z.); rr1272@msstate.edu (R.R.); 2 Tyson Foods, 2200 W. Don Tyson Parkway, Springdale, AR 72762, USA; thu.dinh@tyson.com; 3 Department of Food Science, Nutrition, and Health Promotion, Mississippi State University, Starkville, MS 39762, USA; schilling@foodscience.msstate.edu (M.W.S.);

**Keywords:** aerotolerance, clonal complex, survival, refrigeration, freezing

## Abstract

*Campylobacter jejuni* is one of the leading causes of acute diarrhea in the United States. Despite being a microaerophilic pathogen, *C. jejuni* continues to endure within the domain of food production, especially in poultry processing. Recent research on aerotolerance indicates that close monitoring of this pathogen is necessary. A total of 40 *C. jejuni* isolates previously obtained from commercial broiler processing plants were analyzed for aerotolerance and genetic diversity. In addition, the effect of aerotolerance and storage time (days) on the survival of *C. jejuni* on broiler drumsticks at refrigeration (4 °C) and freezing conditions (−20 °C) was also evaluated. Out of 40 isolates, 25 (62.5%) were aero-sensitive (AS), 10 (25%) were intermediately aerotolerant (IAT), and 5 (12.5%) were hyper aerotolerant (HAT). The isolates belonged to four clonal complexes (CCs) and six sequence types, with the majority of isolates assigned to the CC–353 clonal complex. *C. jejuni* counts were reduced by 0.40 log CFU/g after 7 days at 4 °C and by 1.50 log CFU/g after 14 days at −20 °C, respectively, irrespective of aerotolerance (*p* < 0.001). At both refrigeration (*p* < 0.013) and freezing (*p* < 0.001), HAT showed greater reductions as compared to AS and IAT. These findings suggest that both refrigeration and freezing reduce *C. jejuni* counts.

## 1. Introduction

*Campylobacter jejuni* is one of the leading causes of gastroenteritis in humans [1]. It is a zoonotic commensal bacterium transmitted to humans [2] that colonizes poultry intestines, which contain low oxygen and a higher core temperature (40 to 42 °C). The presence of *C. jejuni* in poultry intestines leads to the contamination of poultry products during processing, which is the major route of transmission of *C. jejuni* to humans [2]. The incidence of campylobacteriosis has increased by 12% compared with 2015–2017, with an estimated 1.5 million infections annually in the United States [3]. According to the CDC Foodborne Disease Outbreak Surveillance System, from 2010 to 2017, 236 *Campylobacter* foodborne outbreaks were reported accounting for 2381 illnesses [3]. *Campylobacter* infections in humans induce self-limiting gastroenteritis, marked by symptoms such as diarrhea, cramps, fever, and vomiting, and are a major risk factor for Guillain–Barre syndrome, a nervous system disorder affecting peripheral nerves [4]. *C. jejuni* is a fastidious microbe with distinct nutritional requirements, and it has been assumed that it is unable to thrive at high oxygen levels in the atmosphere [5]. However, *C. jejuni* has been recovered from both retail meats and processing plants [6,7,8,9]. The incidence of campylobacteriosis in humans as well as isolation of *C. jejuni* from various aspects of the processing plants [8] and retail meat products [7] suggests that these bacteria have evolved survival strategies so that they can survive in the presence of oxygen.

*C. jejuni* has demonstrated aerotolerance to high-oxygen environments [10,11]. A total of 63% of *C. jejuni* isolates on broiler carcasses, dairy products, and clinical samples are hyper aerotolerant (HAT) in comparison to 37% of aero-sensitive strains (AS) [12]. Hyper aerotolerant strains of *C. jejuni* are common in human infections that have been linked to poultry meat [12,13,14]. Although numerous studies have reported the aerotolerance level of *C. jejuni* isolates from various sources (chicken, humans, and dairy products) [12,15], there are limited studies that have reported the level of aerotolerance among isolates from commercial broiler processing plants.

*C. jejuni* is a diverse species with a wide range of genotypes and phenotypes. There are several techniques such as pulsed-field gel electrophoresis (PFGE), amplified fragment length polymorphism (AFLP), and recently multi-locus sequence typing (MLST), used to detect genotypes [16]. MLST measures variations in housekeeping genes of the genome and assigns a unique sequence type to it [17]. Although there are several MLST studies on *C. jejuni*, most of them are concentrated on human isolates [18], isolates from farm environments and live birds [19]. Studies are lacking on the genetic variations among isolates from commercial processing plants. With the increasing level of aerotolerance and human campylobacteriosis cases, the need to understand the level of aerotolerance as well as the genetic diversity of *C. jejuni* that are isolated from poultry processing plants is apparent.

Refrigeration and freezing are crucial for the storage and distribution of safe and high-quality chicken meat. It is also essential to limit the growth and survival of pathogenic and spoilage microorganisms. The ability of pathogenic bacteria like *C. jejuni* to tolerate cold stress plays a significant role in food safety. Although *C. jejuni* requires 37 to 42 °C to grow, it persists in cold poultry processing and storage environments. Based on this observation, the survivability of *C. jejuni* under cold stress may be underestimated since *C. jejuni* has been shown to tolerate refrigeration stress and survive [12]. Aerotolerant strains have been shown to survive longer in refrigeration compared to aero-sensitive ones [12,15]; however, these studies used a *C. jejuni* suspension in a 96-well plate that was stored at 4 °C for 7 days. Moreover, limited data are available on the relationship between the aerotolerance of *C. jejuni* and its cold tolerance in poultry meat. Therefore, it is important to investigate if there is a difference in the survival of aerotolerant and aero-sensitive strains when present on chicken meat at refrigerated (4 °C) and frozen temperatures (−20 °C).

Therefore, the objectives of this study were to investigate the level of aerotolerance and genetic variations among *C. jejuni* populations that were isolated from different commercial broiler processing plants and to analyze the impact of refrigeration and freezing on the survivability of *C. jejuni* on chicken meat. This will give us a better understanding of the level of aerotolerance, genetic variations, and refrigeration tolerance among isolates from poultry processing plants.

## 2. Materials and Methods

### 2.1. Campylobacter Strains and Culture Conditions

A total of 40 *C. jejuni* strains previously collected from various commercial poultry processing plants were used in this study [20]. Isolates were collected from three commercial processing plants from different stages of processing including Mechanically separated meat (MDM), Post pick (PP), Pre-chill (PRC), and Drumstick (DRUM) (Table 1) [20,21]. Isolates were confirmed to be *C. jejuni* using PCR [20] and then stored at –80 °C in 20% glycerol until further use.

### 2.2. Evaluation of Aerotolerance

The aerotolerance of *C. jejuni* strains was determined as previously described with slight modifications [12]. The frozen stock cultures were streaked on a selective *Campylobacter* agar base and incubated in microaerophilic conditions at 42 °C for 48 h. The *Campylobacter* selective agar contained *Campylobacter* agar base (Oxoid, CM0689) that was supplemented with antibiotics (Oxoid, SR0204E) and 5% laked horse blood (Oxoid Ltd., Basingstoke, Hants, UK). Each isolate had three independent replicates. Individual colonies were picked and cultured in 10 mL of brain heart infusion (BHI) broth for 18 h at 42 °C under microaerophilic conditions. Working cultures of each isolate were prepared by serial dilution to approximately 5 log CFU/mL and incubated under aerobic conditions with shaking at 42° and 200 rpm. Bacterial numbers were enumerated at 0, 12, and 24 h by plating onto *Campylobacter* selective agar plates. *C. jejuni* isolates that did not survive the aerobic incubation conditions for 12 h were categorized as aero-sensitive (AS), the isolates that survived for 12 h but did not survive 24 h of aerobic exposure were categorized as intermediate aerotolerant (IAT), and those that survived for more than 24 h under aerobic incubation were categorized as hyper aerotolerant (HAT). As a control group, a similar experiment was performed using the same isolates under microaerophilic conditions (6% oxygen), using an Anoxomat™ Mark II (Spiral Biotech Inc., Norwood, MA, USA).

### 2.3. Multi-Locus Sequence Typing

#### 2.3.1. DNA Extraction

The bacterial DNA was extracted as previously described [20]. Briefly, *C. jejuni* was grown for 18 h in 10 mL of BHI broth and centrifuged to obtain the cell pellet using an Eppendorf Centrifuge 5810 R (Eppendorf, Hamburg, Germany) at 4000 rpm for 10 min. Pellets were washed with PBS, and DNA was extracted using the Thermo Fisher GenJet Genomic DNA purification kit K0721 (Thermo Fisher Scientific, Waltham, MA, USA) according to the manufacturer’s instructions. Purity was evaluated using a NanoDrop One (Thermo Fisher Scientific, Wilmington, DE, USA).

#### 2.3.2. PCR Amplification

PCR amplification was carried out following a *C. jejuni* published protocol in PubMLST [17]. The housekeeping genes *aspA*, *glnA*, *gltA*, *glyA*, *pgm*, *tkt*, and *uncA* were amplified using specific primers (Table 2). In brief, 30 μL PCR mix was prepared for each sample, which included 11 μL of molecular biology grade water, 15 μL of 2X Thermo Scientific™ Phusion Hot Start II High-Fidelity PCR Master Mix, 1.5 μL of 10 μmol l^−1^ each forward and reverse primers, 1 μL of 20 ng/μL of genomic DNA. The amplification conditions were initial denaturation at 98 °C for 30 s followed by 35 cycles of denaturation at 98 °C for 30 s, annealing at 55 to 65 °C, depending upon the Tm of primers, for 30 s, and extension at 72 °C for 30 s, with a final extension at 72 °C for 5 min. The housekeeping genes amplified from genomic DNA from *C. jejuni* ATCC 29428 were used as a positive control. The PCR master mix with DNA elution buffer served as a negative control. The PCR products were separated on 1% agarose gel containing SYBR™ Safe DNA gel stain and visualized under UV light using a Kodak Gel Logic 200 Imaging System (Eastman Kodak Co., Rochester, NY, USA). Amplified PCR products were purified using DNA Clean and Concentrator–5 (ZYMO Research Corporation, Irvine, CA, USA) following the manufacturer’s instructions. Briefly, 25 μL of an amplified PCR product was mixed with 125 μL of DNA binding buffer to give a total volume of 150 μL in a PCR tube. The mixture was then transferred to a Zymo–spin column (ZYMO Research Corporation, Irvine, CA, USA). The column with bound DNA was washed twice with wash buffer, and purified PCR products were eluted with 10 μL of elution buffer. The purified PCR product concentration was measured using NanoDrop One (Thermo Fisher Scientific, Wilmington, DE, USA) and normalized with molecular biology grade water to a concentration of 50 ng/µL. For sequencing, 5 µL of each normalized PCR product for a specific gene was mixed with 5 µL of 2 pmol/µL specific sequencing primers listed in Table 3 to reach a total volume of 10 µL. The pre-mixed samples were then sent to Eurofins Genomic to perform Sanger sequencing (Eurofins Scientific, Louisville, KY, USA).

#### 2.3.3. MLST Allele, Sequence Type (ST), and Clonal Complex (CC) Assignment

All allelic sequences of the seven housekeeping genes were evaluated in the *Campylobacter* MLST database [22]. MLST alleles, STs, and CCs were assigned. Novel allelic combinations that were not assigned to any existing STs in the MLST database were submitted to the *Campylobacter* MLST database to assign new STs and CCs.

#### 2.3.4. Phylogenetic Relationship between *C. jejuni* Isolates

The genotypic relatedness of *C. jejuni* sequence types (STs) with novel STs identified was investigated using the unweighted pair group method in conjunction with the arithmetic mean (UPGMA) method. A UPGMA dendrogram was constructed using the bootstrap test (1000 replicates). The tree is drawn to scale, with branch lengths in the same units as the evolutionary distances that were used to infer the tree. The evolutionary distances were calculated using the p-distance method and were measured in base differences per location. Molecular Evolutionary Genetics Analysis (MEGA11) was used to perform evolutionary analysis.

### 2.4. Effect of Refrigeration and Freezing on C. jejuni Survival on Chicken Drumsticks

Three isolates of *C. jejuni* from each aerotolerance category (Table 4) were streaked on selective *Campylobacter* agar and incubated at 42 °C for 48 h under microaerophilic incubation to obtain individual colonies. A single colony was picked and placed in 10 mL of fresh BHI broth and incubated for 18 h at 42 °C under microaerophilic conditions. After incubation, each culture was vortexed, and serially diluted to reach approximately 7 log CFU/mL. Three *C. jejuni* working culture cocktails were prepared. Each working culture contained three isolates of either AS, IAT, or HAT categories to better simulate the natural environment.

Broiler chicken drumsticks with skin from a retail store were used for this experiment. Two separate studies were conducted, one under refrigeration (4 °C) for 7 days and the other under freezing (−20 °C) for 14 days. Each drumstick (*n* = 135 for refrigeration; *n* = 108 for freezing study) was weighed (~150 g), inoculated with 0.1 mL of working culture cocktails (7 Log CFU/mL) by spot pipetting across the entire drumstick skin surface in a whirl-pak bag to get the natural contamination level. They were then placed in a biosafety cabinet for 45 min to allow for bacteria attachment and stored at the assigned temperature. A total of 15 uninoculated drumsticks were used as negative controls for the refrigeration study and 12 were used for the freezing study, out of which 3 drumsticks were processed on each sampling day. Each study was replicated with three independently prepared culture cocktails, and each cocktail was applied on three drumsticks. *C. jejuni* counts were enumerated on d 0, 1, 3, 5, and 7 when stored at 4 °C and on d 0, 3, 7, and 14 when stored at −20 °C. Drumsticks were rinsed for 1 min in a whirl-pak bag by adding 100 mL of buffered peptone water (BPW). Rinsate from each drumstick was serially diluted, followed by direct plating on *Campylobacter* selective agar plates. The plates were then incubated for 48 h under microaerophilic conditions. The difference in Log CFU/g from d 0 was calculated.

### 2.5. Statistical Analysis

For the aerotolerance assay, means and standard deviation were calculated from three independent replications. Isolates were categorized into three groups consisting of AS, IAT, and HAT and the results were presented in percentages.

For MLST, sequences were analyzed using the *Campylobacter* PubMLST database, and clonal complexes were assigned.

For the refrigeration and freezing study, data were analyzed as a split-plot design. The aerotolerance category was used as the main plot unit and days of storage as the split plot unit. Data were analyzed using SAS 9.4, and significance was considered at *p* < 0.05. Means were separated using the LS-means procedure using the protected *t*-test. The differences in means were considered statistically significant at *p* < 0.05. 

## 3. Results

### 3.1. Aerotolerance Level of C. jejuni Isolates

**Figure 1:**
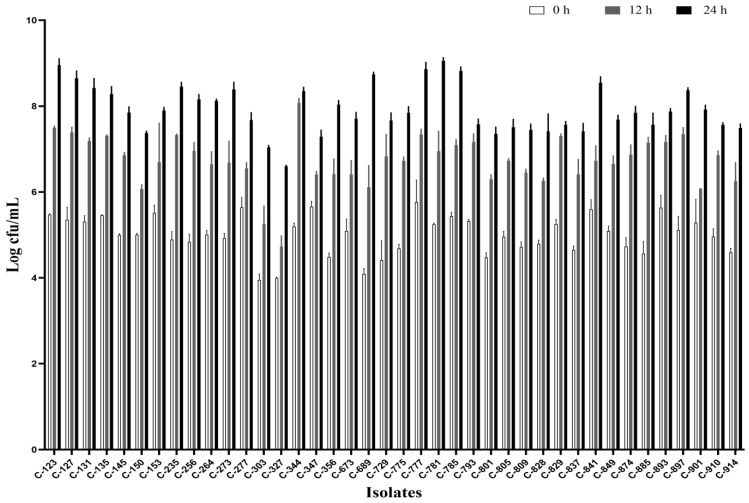
Average Log CFU/ml of *C. jejuni* isolates at 24 h when incubated micro-aerobically at 42 °C in BHI broth with shaking at 200 rpm. The results show the mean and standard deviation of the three independent replicates.

**Figure 2:**
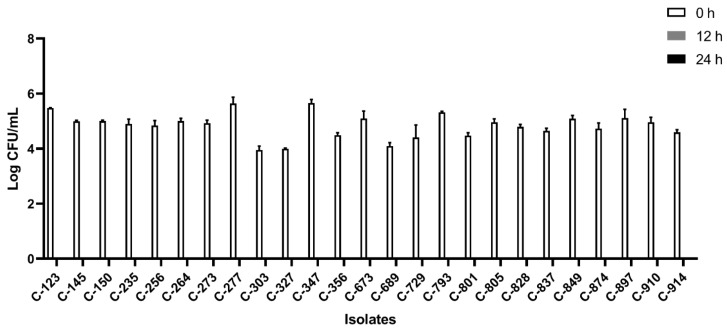
Average Log CFU/ml of aero-sensitive *C. jejuni* isolates at 0 h, 12 h, and 24 h time points in an aerobic condition. The results show the mean and standard deviation of the three independent replicates. These isolates did not survive 12 h and 24 h of aerobic exposure.

**Figure 3:**
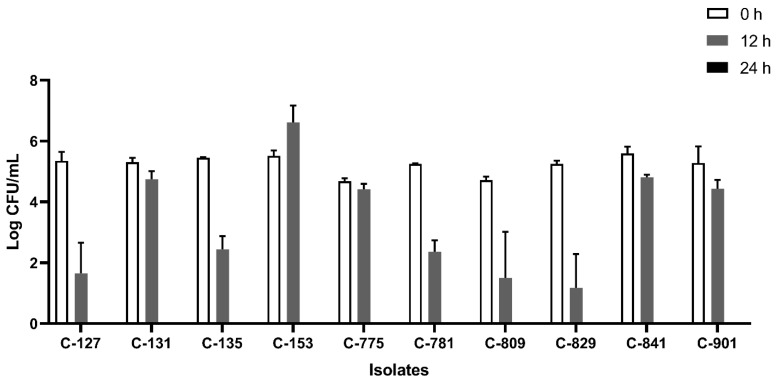
Average Log CFU/ml of intermediate aerotolerant *C. jejuni* isolates at 0 h, 12 h, and 24 h time points in an aerobic condition. These isolates did not survive 24 h of aerobic exposure. The results show the mean and standard deviation of the three independent replicates.

**Figure 4:**
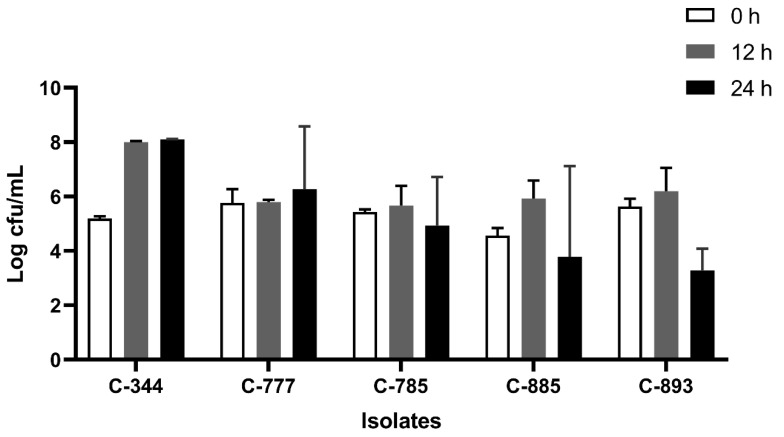
Average Log CFU/ml of hyper aerotolerant *C. jejuni* isolates at 0 h, 12 h, and 24 h time points in an aerobic condition. The results show the mean and standard deviation of the three independent replicates.

All the isolates were able to grow well in microaerophilic conditions with an increase of up to 9 log CFU/mL on 24 h of incubation (Figure 1). Out of 40 isolates, 25 (62.5%) were AS (Figure 2), 10 (25%) were IAT (Figure 3), and 5 (12.5%) were HAT (Figure 4). This result indicates that IAT and HAT isolates are more prevalent in commercial broiler processing than previously expected.

### 3.2. MLST Analysis of C. jejuni Isolates

Out of the 40 *C. jejuni* isolates that were sequenced and analyzed by MLST, 8 sequence types (STs) and 4 Clonal complexes (CCs) were identified. Three of the screened *C. jejuni* isolates did not show any defined sequence type. Those novel sequences and ST were submitted to the *Campylobacter* MLST database. The predominant sequence type was ST–10578 (*n* = 15, 37.5%), followed by ST–2132 (*n* = 10, 25%) which belongs to the same clonal complex as CC–353. The sequence type and their clonal complexes along with the number of isolates are given in Table 5. The dominant CCs in PP isolates were CC–353 and CC–21. The MDM isolates were evenly distributed among CC–353 and CC–21. The PRC isolates were distributed in all CCs except CC–21 (Table 5). The two drumstick isolates belonged to CC–353. These results indicate that there is no distinct pattern in the variation of CCs among isolates from broiler meat at different stages of commercial poultry processing plants. However, there is a pattern of occurrence of certain CCs according to the location of the processing plant. The dominant CC in plant 1 was CC–443 and CC–21 whereas in plant 2, the dominant CC was CC–353. All the isolates from plant 3 belonged to CC–353 as shown in Table 6.

### 3.3. Aerotolerance and Genetic Relatedness

**Figure 5:**
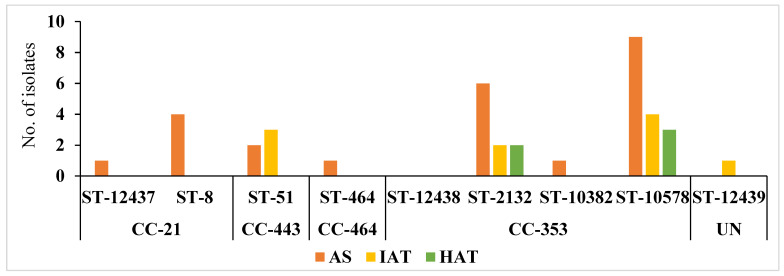
Frequency distribution of *C. jejuni* sequence type (ST) and clonal complexes (CC) among isolates with different aerotolerance levels. AS = Aero-sensitive; IAT = intermediate aerotolerant; HAT = Hyper aerotolerant; UN = Unassigned, not assigned to any clonal complexes.

**Figure 6:**
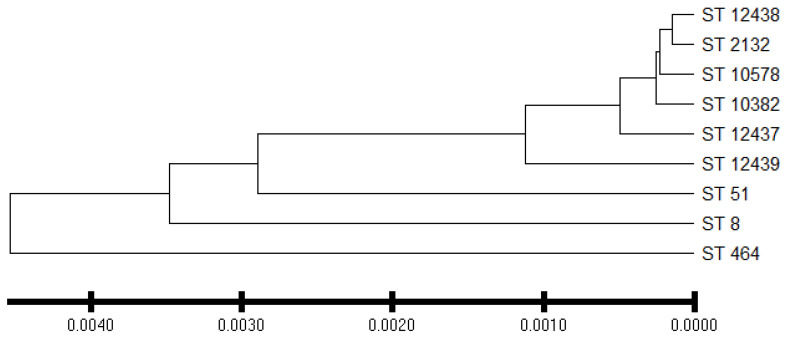
Dendrogram showing phylogenetic relationship between sequence type and isolates whose sequence type was novel. ST = sequence type. ST–12437, ST–12438, and ST–12439 are the new sequence types assigned by the *Campylobacter* PubMLST database. Evolutionary distances were computed using the p-distance method using the UPGMA method.

*C. jejuni* isolates from the commercial broiler processing plant belonged to 4 CCs and 8 STs. AS were predominant in all clonal complexes whereas IAT isolates were detected in CC–353 and CC–443 clonal complexes. HAT predominantly belonged to CC–353 (Figure 5). The relatedness of 3 novel isolates to the clonal complexes identified in our study has been shown in Figure 6.

### 3.4. Refrigeration and Freezing

**Figure 7:**
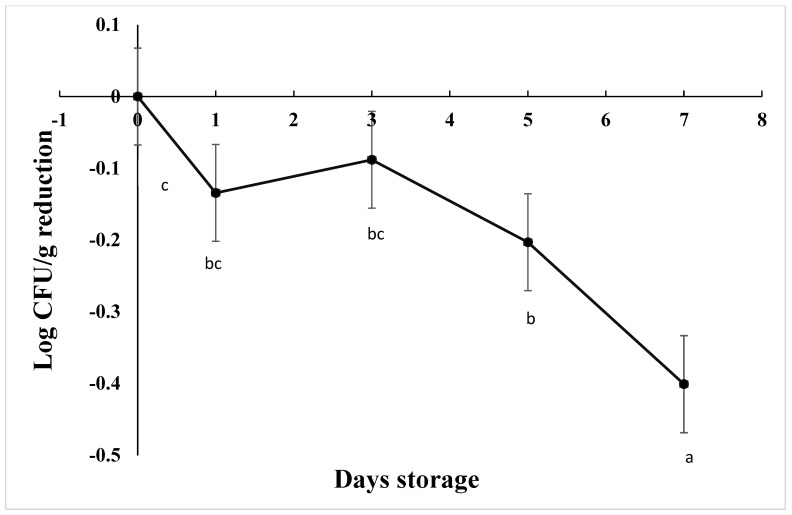
Reduction in *C. jejuni* counts when stored at 4 °C for 7 days (*p* < 0.001). Means with different letters are statistically different. The error bar represents the pooled standard error of the means.

The starting load of *C. jejuni* after inoculation in chicken drumsticks was approximately 3.7 log CFU/g in AS, IAT, and HAT. The number of viable cells was significantly reduced by 0.20 Log CFU/g on d 5 and 0.40 Log CFU/g over 7 days at 4 C (Figure 7), irrespective of aerotolerance (*p* < 0.001). HAT showed greater reductions as compared to AS and IAT at 0.27, 0.14, and 0.08 log CFU/g, respectively (*p* = 0.013). For each treatment and replication, no colonies were detected from negative controls.

**Figure 8:**
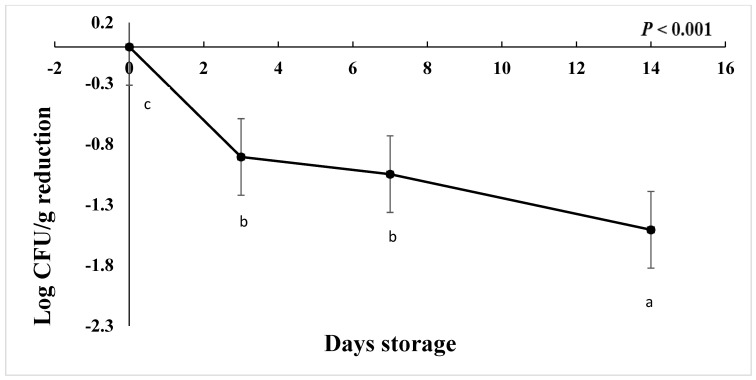
Reduction in *C. jejuni* counts when stored at −20 °C for 14 days (*p* < 0.001). Means with different letters are statistically different. The error bar represents the pooled standard error of the means.

The starting load of *C. jejuni* after inoculation in chicken drumsticks for the freezing study was approximately 3 to 4.7 log CFU/g in AS, IAT, and HAT. The number of *C. jejuni* at ࢤ20 °C was significantly reduced by 1 Log CFU/g on d 3 and d 7 followed by 1.50 log CFU/g on d 14 (Figure 8), regardless of aerotolerance (*p* < 0.001). HAT, AS, and IAT categories were reduced by 1.12, 0.90, and 0.60 log CFU/g, respectively (*p* < 0.001), irrespective of storage time. This shows that IAT is slightly more tolerant to freezing than AS and HAT. No colonies were detected in negative controls from given treatment and replications.

## 4. Discussion

Historically, *Campylobacter* has been reported as a microaerophilic pathogen that was thought to be sensitive to high oxygen in the surrounding environment. Contradictorily, our research showed that aerotolerant isolates are common in poultry processing facilities. In our study, the aerotolerance level of 40 *C. jejuni* isolates were evaluated that were isolated from commercial broiler processing facilities. A greater incidence of hyper aerotolerant strains (12.5%) and intermediate aerotolerant strains (25%) was found in our study. This differs from research on chicken liver and chicken meat isolates where only 6.6% were aerotolerant and 2.6% were hyper aerotolerant [23]. In contrast, the prevalence of HAT was 53.5% and aerotolerant was 38% among *C. jejuni* isolates from human clinical cases [24]. Similarly, research from Japan indicates that 40% of *C. jejuni* isolates (poultry, cattle, and humans) were HAT [25]. Additionally, a study conducted in Egypt indicated that 63% of *C. jejuni* isolates (clinical isolates, dairy products, and broiler carcasses) were HAT [12]. This indicates that *Campylobacter* strains are commonly aerotolerant when human clinical cases are involved. This suggests that aerotolerant strains are associated with *Campylobacter-related* human illnesses. From the findings above, the level of aerotolerance may vary between countries, and this could be due to genetic differences between isolates found in various parts of the world. Alternatively, it could also be due to differences in the sample types that were collected for the study.

Multi-locus sequence typing is an efficient technique that can be used to understand *C. jejuni* population structure, evolution, and distinguish between *C. jejuni* isolates from different geographical areas and sources [17]. To our knowledge, this is the first study that has investigated genetic variation among *C. jejuni* isolates from various commercial broiler processing plants. A considerable level of genetic diversity was found in the *C. jejuni* population in our study. Among 40 *C. jejuni* isolates, CC–353 was the predominant CC. CC–353 is a dominant CC among poultry isolates and has been identified in several studies [26,27,28]. The dominant clonal complex may vary between locations, although CC–353 and CC–21 contain the largest number of *C. jejuni* isolates [19,27,28]. CC–21 are mainly associated with human disease isolates of *Campylobacter* in an MLST-based study where isolates of *Campylobacter* were from cases of human campylobacteriosis, livestock, and the environment [17]. *C. jejuni* isolates from 12 outbreaks in the US were subjected to MLST analysis, which revealed that CC–21 is frequently engaged in human epidemics [29]. CC–21 has also been found in retail chicken meat isolates and bovine and ovine isolates [30]. In the present study, only 4 out of 40 isolates belonged to CC–21. This indicates that the presence of clones that are associated with foodborne illness are present in processing plants. However, all CC–21 isolates were aero-sensitive which suggests that CC–21 isolates from our source may not survive in the outside environment because of its aero-sensitivity and may not necessarily contribute to human epidemics. CC–464 and CC–443 are major CCs in human and chicken isolates [22]. CC–443 was also one of the common CCs found in broiler flocks within grower houses [31]. Results also indicate that isolates from different processing plants belonged to different clonal complexes. A similar trend was reported by Colles et al., (2003) where dominant CC varied according to the location from where the sample was collected [19]. Based on the findings of this study and the wide range of results reported in previous studies, dominant clonal complexes vary with location. One possible factor that contributes to variation could be variability in bird flocks coming to the plant. *Campylobacter* is introduced into the processing plant by *Campylobacter-positive* bird flocks; thus, receiving birds from single or multiple farms might differ the diversity of the *C. jejuni* population. Processing procedures can also alter the clonal complex of *C. jejuni.* Processing procedures that allow for cross-contamination between various bird flocks or processing sites, for example, could aid in the spread of specific strains or clones of *C. jejuni*. *C. jejuni* populations differ depending on host species, environmental niches [32], and season [31]. Therefore, the seasonality of the processing prevalence of the bacteria in the environment such as in water and soil might have resulted in differences in clonal complexes as well as the occurrence of a mixed clonal complex in the same processing plant.

All HAT isolates belonged to CC–353. However, this could be because CC–353 is the predominant clone in our study which can be correlated to the source of isolation, as CC–353 predominates in poultry isolates. Interestingly, it is noteworthy that the majority of HAT isolates in a previous study belonged to CC–21 [11]. In contrast, none of the HAT isolates belonged to CC–21, even though the majority of isolates were from CC–21 [32]. This emphasizes the diversity of *C. jejuni* and although the explanation for this discrepancy is unknown, the prevalence of HAT strains and the CC complex it belongs to may be associated with the place of origin and/or the source of isolation.

As part of this study, we evaluated whether there is a difference in the ability of AS, IAT, and HAT isolates to tolerate 4 °C for 7 days and −20 °C for 14 days. *C. jejuni* counts were reduced in all aerotolerance categories, though HAT strains were subject to a greater reduction at both storage conditions. These results differ from a previous study in which IAT and HAT were more tolerant compared to AS when incubated for 7 days at 4 °C and −20 °C [15]. Similarly, another study showed that HAT is more tolerant to refrigeration and freezing than AS and IAT [12]. However, these discrepancies may be due to experimental conditions as those studies were conducted in vitro in a 96-well plate. The meat matrix can establish microenvironments that influence bacterial growth and survival, and these circumstances can differ in vitro and in vivo. It has been reported that the pH of meat plays a significant role in bacterial survival [33,34]. The pH of the chicken drumstick (5.8–6.2), for example, may differ from those used in the in vitro investigation (MH broth pH ~ 7.4), resulting in variances in the bacterial survival rate. Interestingly, in our study, all AS, IAT, and HAT survived for 7 days under refrigeration. However, when aero-sensitive and aerotolerant strains were inoculated in the raw poultry meat under aerobic conditions, aero-sensitive isolates survived for 3 days whereas aerotolerant isolates survived until 14 days [35], which indicates that aerotolerant isolates were more tolerant to refrigeration. Though previous studies [12,15,35], found that aerotolerance significantly affected the viability of *C. jejuni* under cold stress, the observation was not the same in our study. Both refrigeration and freezing storage reduced overall *C. jejuni* counts over the days in our study. The reduction was 0.4 log CFU/g after 1 week of refrigeration, whereas for freezing, the reduction was almost 1 log CFU/g during the first week and 1.51 Log CFU/g during the second week. Our results were in line with a previous study [36] which reported that one week of freezing at −20 °C decreased *C. jejuni* in chicken meat significantly, with further decreases reported after two weeks. This shows that cold storage may reduce the *C. jejuni* counts on poultry meat, but the rate of reduction may depend on temperature and storage time. Therefore, keeping poultry meat at cold temperatures is important for minimizing the amount of *C. jejuni*, which can contribute to lowering the incidence of campylobacteriosis in people.

## 5. Conclusions

In conclusion, there was a genetic diversity among *C. jejuni* that were isolated from commercial broiler processing plants. Furthermore, it was determined that aerotolerant isolates are prevalent in processing plants and the level of aerotolerance did not affect the cold tolerance in *C. jejuni*. Refrigeration and freezing reduced *C. jejuni* counts in chicken drumsticks. While refrigeration and freezing *C. jejuni*-contaminated samples is not a cure-all, it can lower the proliferation of *Campylobacter* in meat and reduce the chances of human campylobacteriosis.

Overall, our findings provide an insight into the distribution of different clonal complexes among different processing plants even though they fall in similar geographical locations (Southeastern United States). One limitation of this study is that we only utilized 40 *C. jejuni* isolates, therefore we cannot make a broad conclusion on genetic variations of *C. jejuni* isolates in the processing plants. Moving forward, it will be interesting to analyze isolates from different processing plants from various geographical locations and provide further insight into the population structure of *C. jejuni* in processing facilities. Also, because campylobacteriosis is a frequently reported food-borne illness, and results indicate that refrigeration and freezing decrease the *C. jejuni* counts in chicken drumsticks, it would be interesting to see if *C. jejuni* is dying or just converting into the viable non-culturable state as a response to cold stress.

## Figures and Tables

**Table 1:** Sources and number of *C. jejuni* isolates from different steps of commercial broiler processing plants.

Source	No. of *C. jejuni* Isolates
Mechanically separated meat (MDM)	8
Post pick (PP)	11
Pre-chill (PRC)	19
Drumstick (DRUM)	2

**Table 2:** Primer sets used for polymerase chain reaction (PCR) of the seven housekeeping genes used in the multi-locus sequence typing (MLST) scheme for *C. jejuni*.

Gene	Primer Sequence (5’–3’)	Product Size	Reference
*aspA*	AGTACTAATGATGCTTAT CC	941	[17]
	ATTTCATCAATTTGTTCTTTGC		
*glnA*	TAGGAACTTGGCATCATATTACC	1305	[17]
	TTGGACGAGCTTCTACTGGC		
*gltA*	CCAAATAAAGTTGTCTTGGACGG	1112	[17]
	GGGCTTGACTTCTACAGCTAC TTG		
*glyA*	GAGTTAGAGCGTCAATGTGAAGG	1052	[17]
	AAACCTCTGGCAGTAAGGGC		
*pgm*	TACTAATAATATCTTAGTAGG	1195	[17]
	CACAACATTTTTCATTTCTTTTTC		
*tkt*	AAAGCATTGTTAATGGCTGC	1133	[17]
	GCAAACTCAGGACACCCAGG		
*uncA*	ATGGACTTAAGAATATTATGGC	1259	[17]
	ATAAATTCCATCTTCAAATTCC		

**Table 3:** Primer sets used for sequencing of the seven housekeeping genes to perform multi-locus sequence typing (MLST) of *C. jejuni*.

Gene	Primer Sequence (5’–3’)	Reference
*aspA*	AAGCGCAATATCAGCCACTC	Unpublished
*glnA*	TAGGAACTTGGCATCATATTACC	Unpublished
*gltA*	CCAAAGCGCACCAATACCTG	[17]
*glyA*	AGGTGATTATCCGTTCCATCGC	[17]
*pgm*	TCCAGAATAGCGAAATAAGG	[17]
*tkt*	ACTTCTTCACCCAAAGGTGCG	[17]
*uncA*	ATTCTTTGTCCACGTTCAAG	Unpublished

**Table 4:** Isolates along with their clonal complex used from each aerotolerance category to make cocktails for inoculation in chicken drumsticks for the refrigeration and freezing study.

Aero-Sensitive	Intermediate Aerotolerant	Hyper Aerotolerant
C–256 (CC–21)	C–153 (CC– 443)	C–785 (CC–353)
C–273 (CC–21)	C–127 (CC–443)	C–893 (CC–353)
C–264 (CC–21)	C–135 (CC–443)	C–777 (CC–353)

**Table 5:** Clonal complex (CC) and sequence type (ST) of *C. jejuni* isolates from different sources from the broiler processing plant identified by multi-locus sequence typing (MLST).

Clonal Complex (No. of Isolates)	Sequence Type	Source				Total
		Drumstick	MDM	PP	PRC	
CC–353 (27)	ST–2132	1	–	3	6	10
	ST–10382	–	–	1	–	1
	ST–10578	1	4	6	4	15
	ST–12438	–	–	–	1	1
CC–21(5)	ST–8	–	4	–	–	4
	ST–12437	–	–	–	1	1
CC–464(1)	ST–464	–	–	–	1	1
CC–443(6)	ST–51	–	–	–	6	6
UN (1)	ST–12439	–	–	1	–	1

CC, clonal complex; ST, sequence type; MDM, mechanically separated chicken; PP, post pick; PRC, pre-chill; UN, unassigned.

**Table 6:** Number of isolates from each broiler processing plant and major clonal complex identified in each processing plant.

Processing Plants	Number of Isolates	Major Clonal Complex (No. of Isolates)
Plant–1	12	CC–443 (6); CC–21 (5); CC–353 (1)
Plant–2	5	CC–353 (4); CC–464 (1)
Plant–3	23	CC–353 (22); UN (1)

CC, clonal complex; UN, unassigned.

## Data Availability

The data used to support the findings of this study can be made available by the corresponding author upon request.
